# EEA for sellar chodrosarcomas: case series with literature review

**DOI:** 10.1186/s41016-025-00397-4

**Published:** 2025-06-26

**Authors:** GuoFo Ma, Ning Qiao, Wentao Wu, BoChao Zhang, Kefan Cai, SongBai Gui

**Affiliations:** 1 Department of Neurosurgery, Peking University Third Hospital, Peking University, Beijing, 100191 China; 2 Department of Neurosurgery, Beijing Tiantan Hospital, Capital Medical University, Beijing, China

**Keywords:** Sellar, Chondrosarcomas, Differential diagnosis, Radiological features, Endocrine disorders

## Abstract

**Background:**

Chondrosarcoma is a rare and malignant tumor in the sellar region. Due to the limited understanding of its oncological behavior, it is often misdiagnosed as other lesions, such as chordoma or invasive pituitary adenoma. In the past, craniotomy was considered the primary treatment option. However, with the advancement of neuroendoscopic techniques, many centers have begun adopting endoscopic approaches for this disease. In this article, we summarized our case series and reviewed the previous papers to evaluate the clinical outcomes of neuroendoscopic resection for sellar chondrosarcomas.

**Methods:**

Four patients with sellar chondrosarcomas who underwent tumor resection by endonasal endoscopic approach (EEA) in our institute from 2017 to 2021 were reviewed. In addition, we reviewed the current literatures on sellar chondrosarcomas.

**Results:**

Our series included 4 patients, and 8 cases of sellar chondrosarcomas were reported in previous literatures. In the pooled cohort, there were 6 males and 6 females. The median age at diagnosis was 28.5 years (interquartile range, 22.8–39.3). The most common clinical presentation was blurring of vision (66.7%) and headache (50%). The incidence of preoperative endocrine disorder in such patients was 33.3%; these abnormalities could return to normal after surgery. Complete resection and incomplete resection were achieved in 7 (58.3%) and 5 (41.7%) cases, respectively. Among the 12 patients, only one patient was diagnosed with chondrosarcoma preoperatively; other patients were misdiagnosed with chordoma (*n* = 5; 41.7%), invasive non-functioning pituitary adenoma (INPA) (*n* = 4; 33.3%), or craniopharyngioma (*n* = 2; 16.7%).

**Conclusions:**

The preoperative diagnosis of sellar chondrosarcoma remains challenging and often requires differentiation from chordoma, INPA, or craniopharyngioma. When a calcified mass in the sellar region presents with intact or slightly disturbed anterior pituitary function, heterogeneous enhancement and no diffusion restriction on Magnetic Resonance Imaging(MRI) sequences, and surrounding bony destruction and bony attachment on Computed Tomography(CT) scans, a chondrosarcoma should be suspected preferentially. Complete resection is the optimal goal for the management of sellar chondrosarcoma, but adjuvant radiotherapy and periodic follow-up should be highlighted.

## Background

 Intracranial chondrosarcomas are rare and malignant tumor entities, comprising approximately 0.2% of all intracranial tumors and 6% of skull base tumors [1, 2]. They most commonly occur in the petroclival region arising from the degenerated chondrocytes within the synchondrosis. Headaches, cranial neuropathies and invasion of the hypothalamus are the main complaints. Historically, the traditional transcranial approaches are the main treatment options. In the last decade, however, EEA has become increasingly popular for exposure and resection of these tumors [3, 4].Today, the best treatment for these tumours remains a matter of debate. The surgical goal is to offer a higher rate of resection, meanwhile with the lower post-operative morbidity and mortality. The endoscopic technique, developed in the last 15 years, has achieved equal or better extents of resection and in addition has been associated with a lower rate of complications.

Chondrosarcomas occurring in the sellar region are rare and frequently misdiagnosed as other common sellar lesions, such as pituitary gland tumor, craniopharyngioma, and chordoma. The first case of sellar chondrosarcoma was described by Allan et al. in 2001 [5]. To date, the symptom spectrum, diagnosis, treatment, and prognosis have yet to be well understood. In this study, we reported our 4 patients and integrated the previously published cases to further discuss their presenting symptoms, radiological features, and differential diagnosis.

## Methods

A total of 4 cases of sellar chondrosarcomas who underwent expanded endoscopic endonasal surgery (EEES) between 2017 and 2021 confirmed by histopathological examination and immunohistochemical staining were included in this study. The medical records included clinical as well as operative records and radiographic images. The following clinical and image data were collected, including age at diagnosis, sex, clinical preoperative symptoms as well as duration, hormone level, treatment strategy (extent of resection, postoperative radiation/chemotherapy), and follow-up outcome. We performed using the PubMed queries for articles published in the English language between January 2000 and November 2021 by 2 independent review authors, with the search terms “chodrosarcomas,” “intracranial,” and “saller”. References from reviewed publications were further examined for other studies that met inclusion criteria. Inclusion criteria were articles with PILMS diagnosed by surgical pathology and reports with available clinical data. Eight cases of previously reported sellar chondrosarcomas in the English language were selected. The present study was approved by the Ethics Committee of our hospital.

## Results

**Fig. 1:**
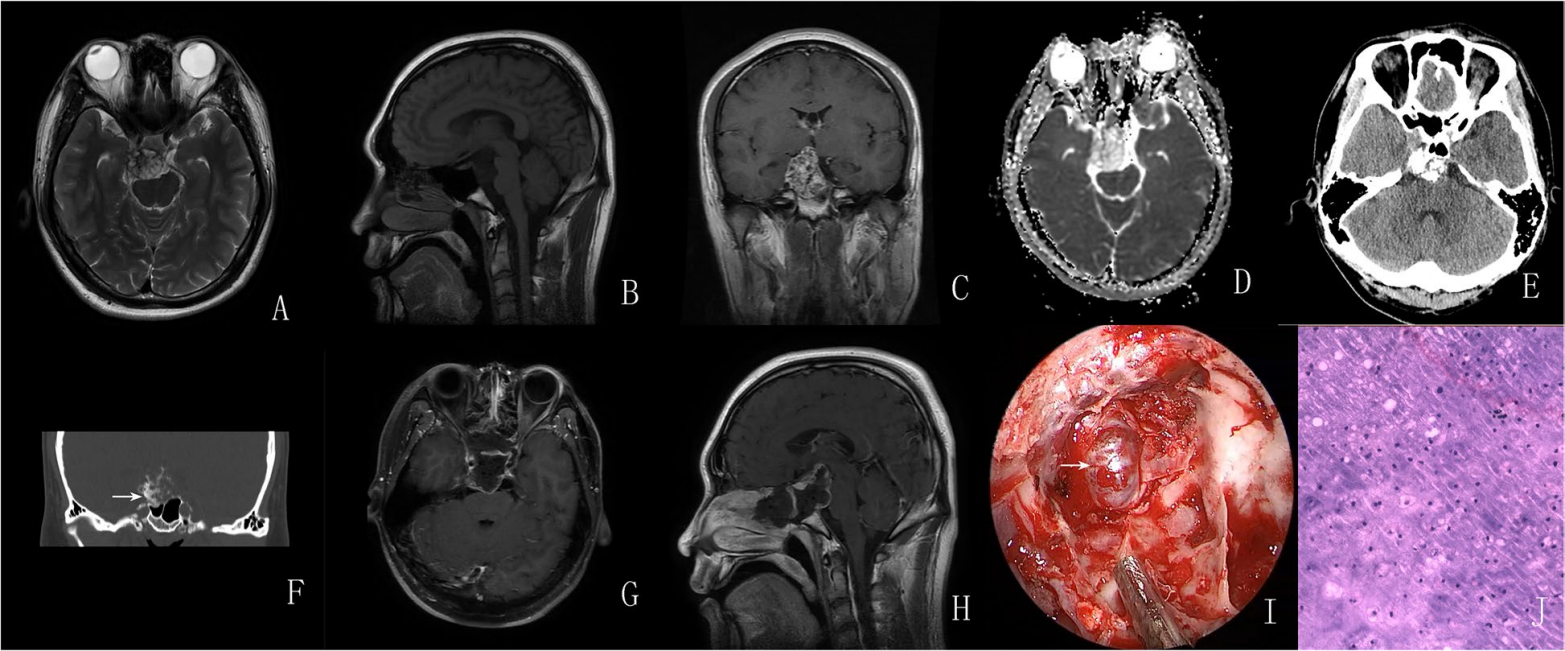
**A**–**C** Preoperative MRI revealed an oval lesion in the intrasellar and suprasellar region with mixed signal intensity on T2-weighted imaging, hypointensity on T1-weighted imaging, and heterogeneous enhancement following the administration of gadolinium. **D** ADC sequence showed no diffusion restriction in the tumor. **E**–**F** CT scan showed that tumor contained an irregular calcified nodule; the bone window setting showed bony destruction and bony attachment (arrow). **G**–**H** Postoperative MRI showed that tumor complete resection was achieved using an endonasal endoscopic approach. I: Intraoperatively, the tumor was extradural with close adhesion to the clivus dura mater and the dura mater was intact (arrow). **J** Pathological examination confirmed the diagnosis of chondrosarcoma with immunopositivity for Vimentin and S-100

**Fig. 2:**
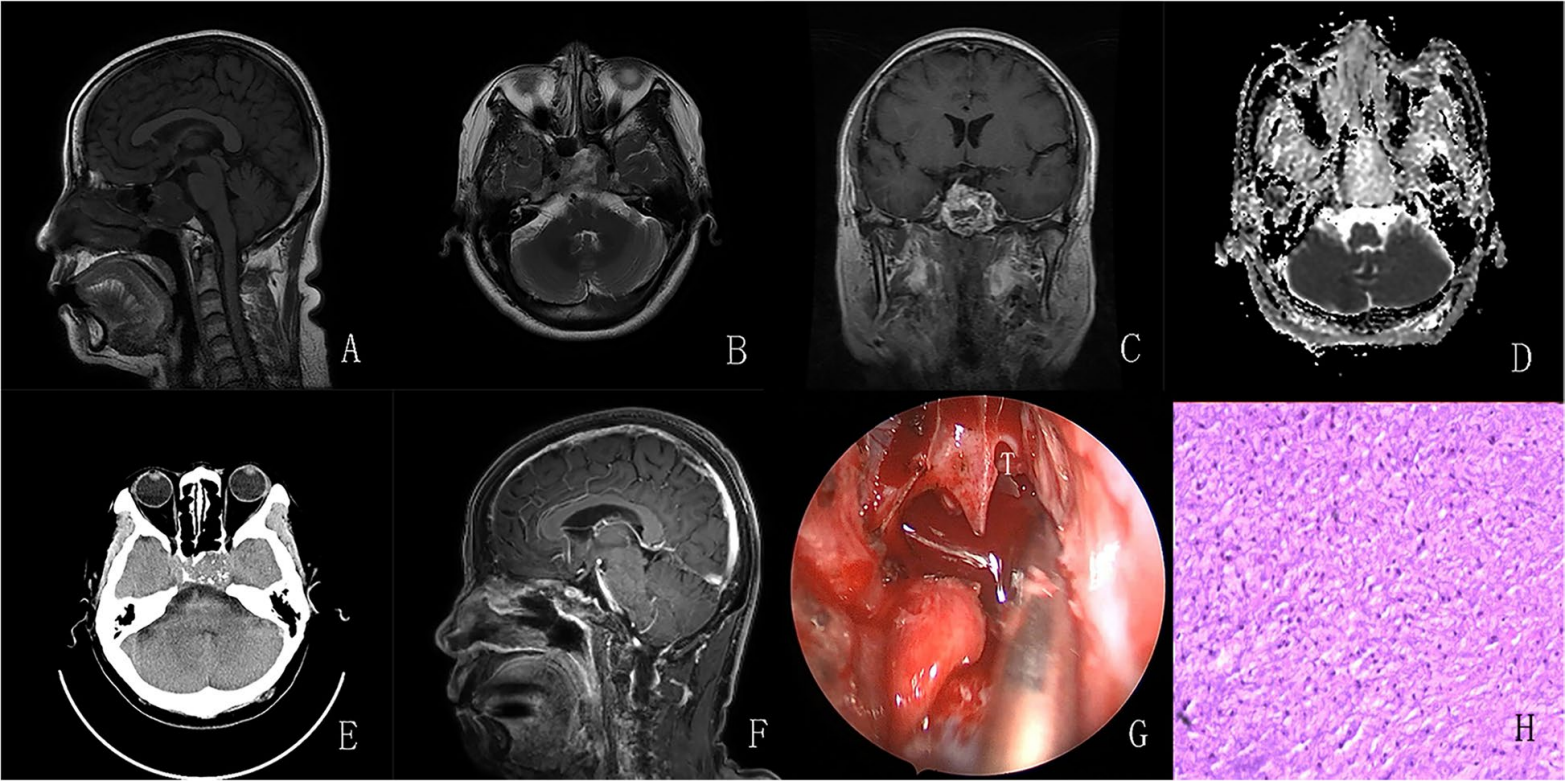
**A**–**C** Preoperative MRI revealed an irregular mass in the sella with the involvement of the sphenoidal sinus and bilateral cavernous sinus. The lesion presented on T1-weighted imaging, hypointense mixed signal intensity on T2-weighted imaging, and heterogeneous enhancement following the administration of gadolinium. **D** ADC sequence showed no diffusion restriction in the tumor. **E** CT scan showed that tumor contained scattered calcification. **F** Subtotal resection was achieved using an endonasal endoscopic approach. **G** The tumor was extended into the sphenoid sinus and bone deficiency was observed in the anterior wall of the sphenoid sinus. T: tumor. **H** Pathologic examination revealed a monomorphic population of tumor cells with strong with Vimentin and S-100 positivity

**Fig. 3:**
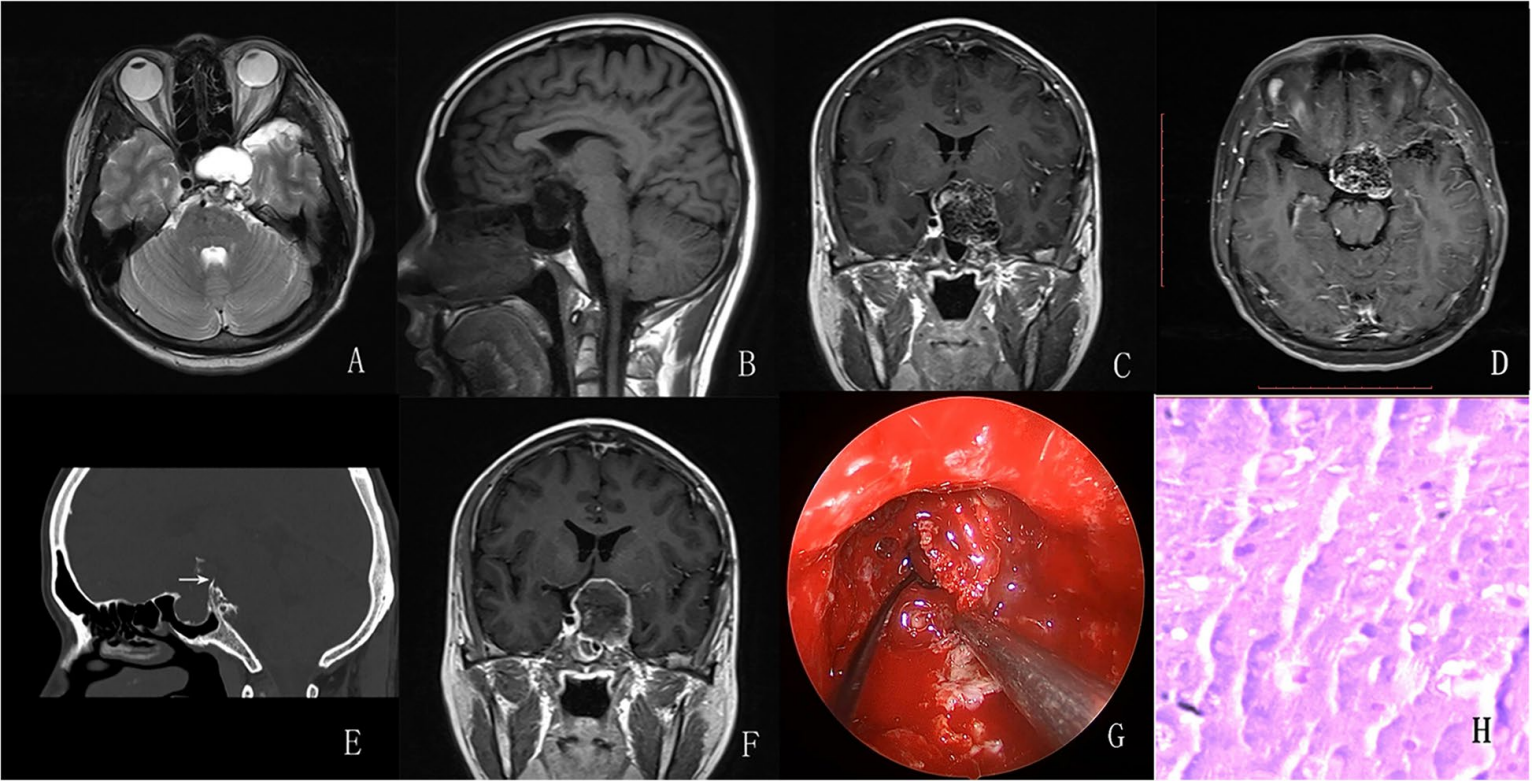
**A**–**D** Preoperative MRI revealed an oval mass in the intrasellar and suprasellar region with the involvement of the sphenoidal sinus and bilateral cavernous sinus with hyperintensity on T2-weighted imaging, hypointensity on T1-weighted imaging, and heterogeneous enhancement following the administration of gadolinium. **E** Bone window setting showed curvilinear calcification and bony attachment (arrow). **F** Tumor complete resection was achieved via endoscopic endonasal surgery. **G** The calcified nodule was removed. **H** HE staining revealed tumor cells with abundant cytoplasm, scattered in a background of myxoid matrix

**Fig. 4:**
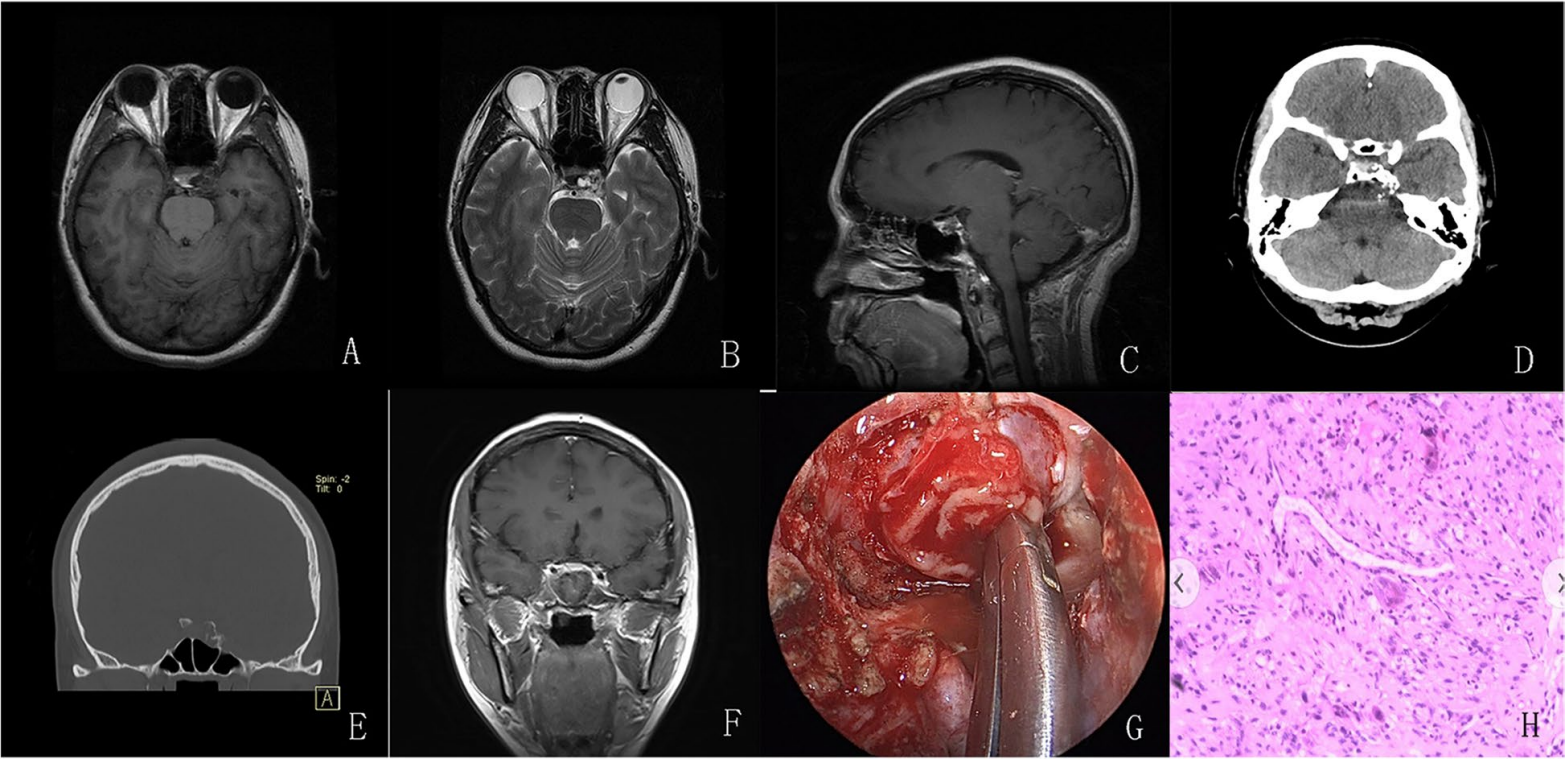
**A**–**C** Preoperative MRI revealed a cystic-solid lesion in the sellar region. The lesion presented mixed intensity on T1- and T2-weighted images, with heterogeneous enhancement observed. **D** An irregular nodular calcification was noted in the tumor. **E** Bone window setting showed curvilinear calcification and bony destruction. **F** Tumor complete resection was achieved via endoscopic endonasal surgery. **G** Intraoperatively, brown liquid of the cystic content was observed. **H** HE staining revealed tumor cells floating in a background of abundant myxoid matrix

The authors described 4 patients with sellar chondrosarcomas treated by using EEES and reviewed 8 cases of previously reported sellar chondrosarcomas in the English language. Their detail data are summarized in Table 1. We performed statistical analysis of data from 12 patients. In the pooled cohort (*n* = 12), the median age was 28.5 years (IQR 22.8–39.3 years), and the sex ratio is 1. The clinical manifestations at diagnosis included blurring of vision (66.7%), headache (50%), diplopia (33.3%), hypogonadism (25%), ptosis (16.7%), and facial sensory disturbance (16.7%). The mean duration of symptoms was 17.09 ± 11.19 months (available duration was not provided for 1 case). Complete resection and incomplete resection were achieved in 7 (58.3%) and 5 (41.7%) cases, respectively. Adjuvant radiotherapy was administered to 5 (41.7%) patients. After a mean follow-up duration of 18.6 ± 14.8 months, 3 patients experienced recurrence, and 1 patient died of tumor progression. Radiologically, the most patients presented hypointensity on T1-weight images, hyperintensity on T2-weighted images, and usually heterogeneous enhancement with the administration of gadolinium (Figs. 1, 2, 3, and 4). In addition, calcified nodule and bony destruction could be observed in all tumors. Among the 12 patients, only one patient was diagnosed with chondrosarcoma preoperatively; other patients were misdiagnosed with chordoma (*n* = 5; 41.7%), invasive non-functioning pituitary adenoma (INPA) (*n* = 4; 33.3%), and craniopharyngioma (*n* = 2; 16.7%) (Table 1).

**Table 1:** Review of reported 8 cases of chondrosarcoma in sellar area and our case series

Authors	Age/sex	Clinical presentations	Preoperative endocrine tests	Preoperative diagnosis	Treatment	EOR	Grade	Radiological features	Follow-up(mons)/recurrence
Zhang Zhen et al. 2019	52/M	Left facial numbness for around 2 years and blurred vision	Normal	Chordoma	Tran + RT	CR	I	Mixed intensity on T1 and T2 images; bone defect on CT scans	12/No
Chang Ding et al. 2019	27/F	Paroxysmal headaches over 1 month and left ptosis for 2 weeks	Normal	Craniopharyngioma	Tran + GKRT	ICR	I	Hypointensity T1 and hyperintensity T2 images; heterogeneous enhancement; significant calcified nodular	N/A
Allan CA et al. 2001	37/F	3 years of headaches and right-sided intermittent retro-orbital pain and 2 years of blurred of vision in the left eye	PRO↑; LH↓	Invasive non-functioning pituitary adenoma	EEA + GKRT	ICR	I	Hypointensity T1 and hyperintensity T2 images; heterogeneous enhancement	18/No
Inenaga C et al. 2003	27/M	Diplopia, right blepharoptosis and facial pain	Normal	Invasive non-functioning pituitary adenoma	EEA + GKRT	ICR	MC	Slight hypo-intensity on T1-weighted images, mixed intensity on T2-weighted images, and heterogeneous enhancement	3/Yes
Junguo Cao et al. 2018	45/F	A 7-month history of amenorrhea and a 3-month history of progressive visual loss in the left eye	PRO↑; LH↓;TSH↓	Invasive non-functioning pituitary adenoma	EEA + Tran (staged approaches + GKRT)	CR	I	Heterogeneous hypointensity on T1-weighted imaging and heterogeneous hyperintensity on T2-weighted imaging; heterogeneous enhancement-like flower ring	14/Yes
Sharma M et al. 2016	40/F	A 1-year history of intermittent headaches and blurring of vision	Normal	Craniopharyngioma	MT + RT	ICR	II	Heterogeneous hypointensity on T1-weighted imaging and heterogeneous hyperintensity on T2-weighted imaging	N/A
Dutta G et al. 2018	22/M	Intermittent headache for over 2 years along with diplopia and diminished visual acuity for the past 3 months	Normal	Pituitary adenoma	Tran + RT	CR	I	Heterogenous signal on T1/T2/FLAIR images with GRE hypointense area with no diffusion restriction; heterogeneous enhancement	N/A
Yuanlong Zhang et al. 2019	20/M	A history of headache for over 3 years and progressive visual blurring for over 1 month	Normal	Chondrosarcoma	EEA	CR	II	Hypointensity T1 and hyperintensity T2 images; heterogeneous enhancement; high signal of DWI	10/No
Present series	20/M	A 1-year history of intermittent headache and a 2-month history of diplopia	Normal	Chordoma	EEA	CR	I	Hypointensity on T1-weighted images and mixed intensity on T2-weighted images; heterogeneous enhancement; high signal of ADC	17/No
	25/F	A 9-month history of irregular menstruation and 5-month history of blurring of vision in the right eye	PRO↑;TSH↓	Chordoma	EEA	ICR	I	Isointensity on T1-weighted images and iso- and hyperintensity on T2- weighted images; heterogeneous enhancement; high signal of ADC	12/No
	30/M	A 1-year history of blurring of vision and sex incompetence	PRO↑; T4↓;T↓; SC↓	Chordoma	EEA	CR	I	Mixed hypointensity on T1-weighted images and hyperintensity on T2- weighted images; heterogeneous enhancement	27/Yes
	36/F	A 4-month history of diplopia. Ophthalmological examination revealed no reduction in visual acuity	Normal	Chordoma	EEA	CR	I	Irregular cystic-solid mass in the sella and left cavernous sinus	54/No

*M *Male, *F *Female, *PRO *Prolactin, *LH *Luteinizing hormone, *TSH *Thyroid-stimulating hormone, *T4 *Thyroxine, *T *Testosterone, *SC *Serum cortisol, *Tran *Transcranial approach, *GKRT *Gamma knife radiotherapy, *RT *Radiotherapy, *EEA *Endoscopic endonasal approach, *MT *Microscope transnasal, *EOR *Extent of resection, *CR *Complete resection, *ICR *Incomplete resection, *MC *Mesenchymal chondrosarcoma, *FLAIR *Fluid-attenuated inversion recovery, *GRE *Gradient echo, *DWI *Diffusion weighted imaging

## Discussion

Most sellar and parasellar tumors arise from the pituitary gland, only approximately10% of lesions are of nonpituitary origin, such as, primary or secondary vascular abnormalities, meningiomas, and bone tumors. Furthermore, roughly 10% of such nonpituitary sellar masses are cartilaginous tumors; chordomas are more common than chondrosarcomas.

## Clinical symptoms, radiological features, and histological characteristic

Sellar chondrosarcomas, similarly with INPA and other non-pituitary sellar tumors, may present with impairment of the visual fields and decreased acuity, signs of hypopituitarism, and nonspecific symptoms such as headache [6]. The most common preoperative symptom was blurring of the vision (*n* = 8; 66.7%), followed by headache (*n* = 6; 50%). Information in previous reports on the endocrine function of patients with such tumors is sparse [7]. We found that although the majority of sellar chondrosarcomas remained epidural growth, they could compress the hypothalamic-pituitary axis resulting in slight hypopituitarism (including hypogonadism, hypocortisolism and hypothyroidism) and elevating the prolactin level. The prevalence of preoperative endocrine dysfunction was 33.3% in the pooled cohort, and these abnormalities could return to normal after surgery.

MRI could provide the exact location, extent of the lesion, and the degree of brain stem compression. Additionally, CT scans allowed identification of calcification and the degree of bony destruction. On MRI scans, Abele et al. concluded that the most typical presentation was moderately low signals on T1-weighted imaging and heterogeneous high signals on T2-weighted imaging with heterogeneous enhancement [8]. In the pooled cases, MRI had mainly hypointensity on T1-weighted images (9 cases (75%)) and mainly hyperintensity on T2-weighted images (12 cases (100%)), with heterogeneous enhancement (12 cases (100%)) detected. Our results were consistent with the previous reports. In 2 patients, the lesions had predominant isointensity on T1-weighted images. In another patient, the lesion presented with partly cystic change, the cystic content was with hyperintense on T1-weighted images and hypointense on T2-weighted images. Preoperatively, the cystic part was suspected to be Rathke’s cleft cyst, whereas which was identified as chondrosarcomas. The data of diffusion-weighted imaging (DWI) and/or apparent diffusion coefficient (ADC) were available in four 4 patients, and no diffusion restriction was observed in any patients. On CT scans, irregular bony destruction and calcification can be noted in all patients, and the soft-tissue component is iso- or hypodense [9].

All sellar chondrosarcomas in our series exhibited similar histological features. The typical manifestation of chondrosarcomas with hematoxylin and eosin (HE) staining was cartilaginous tumor cells that formed a web or strands floating in abundant myxoid matrix (Figs. 1, 2, 3, and 4; 1 J; 2, 3, and 4H). Immunohistochemical investigations were performed using paraffin-embedded tissue samples. Immunohistochemistry revealed negative staining for cytokeratin (CK) and epithelial membrane antigen (EMA) and positive staining for S-100 and Vimentin in our 4 patients. These findings were consistent with a well-differentiated chondrosarcoma [10].

### Differential diagnosis

The sellar lesions are varied, including benign or malignant lesions, vascular abnormalities, and infectious or inflammatory processes [5]. Owing to their overlapping clinical symptoms and anatomic imaging features, the preoperative diagnosis was challenging. However, preoperative differentiation among these entities is important not only for developing therapeutic regimen, but also for prognosis. Our results showed that sellar chondrosarcomas were most commonly misdiagnosed as chordoma, followed by INPA.

Both chondrosarcoma and chordoma are cartilaginous tumors originating from the skull base. They show similar signal intensities on conventional MR sequences and similar enhancement patterns. They also show similar bony destruction and calcified patterns on CT scanning. Additionally, the common symptoms at onset of chordomas are similar to sellar chondrosarcomas and include blurring of vision, headache and diplopia. Therefore, the preoperative differential diagnosis of sellar chondrosarcoma and chordoma is difficult. Interestingly, we found that no diffusion restriction was observed on DWI/ADC sequences of the 4 patients with sellar chondrosarcomas. A possible cause is that chondrosarcoma contains a large amount of myxoid matrix and hyaline cartilage, which can reduce the resistance of diffusion of water molecules. However, the histology of chordoma is characterized by abundant tumor cells, a high ratio of nucleus/cytoplasm and narrow intercellular space. Therefore, the ADC of chondrosarcoma was significantly higher than that of chordoma. In the reports by Müller and Yeom, they also verified the different presentation on DWI/ADC sequences between chordoma and chondrosarcoma. Although the method is not conclusive, which needs to be affirmed in larger series, it provides a potential clue to differentiate the 2 entities.

Clinically, it is not difficult to distinguish invasive prolactinoma from sellar chondrosarcoma with mild hyperprolactinemia by comparing prolactin levels. We found that the prolactin level in patients with sellar chondrosarcoma were significantly lower than those in patients with prolactinomas. When prolactin is elevated greater than 200 ng/mL, a prolactinoma should be considered preferentially. However, the differential diagnosis of sellar chondrosarcoma and INPA is complex. There are no specific differences in tumor locations and clinical presentations between the 2 entities. The most common MRI presentation of INPA is hyperintense signals on T2-weighted images, hypointense signals on T1-weighted images, and homogeneous enhancement after administration of contrast agent. Different enhancement patterns between sellar chondrosarcomas and INPA are helpful in discerning the 2 entities. However, partial INPAs show heterogeneous enhancement due to cystic change, hemorrhage, and necrosis within the tumors [11]. Therefore, it is not always feasible to distinguish the 2 entities by MRI alone. When the heterogeneous post-contrast internal architecture is not that of the typical INPA, further evaluation by CT scanning is necessary. CT scanning can often detect curvilinear calcification, bony destruction, and/or attachment to the skull base. These features, which indicate tumor originating from the skull base, are especially useful in differentiating chondrosarcomas from INPA [11]. Furthermore, the reports on endocrine function in patients with INPA showed that the rates of growth hormone deficiency and hypogonadism were 85% and 75%, respectively, which were significantly higher than in sellar chondrosarcoma [12]. Hence, in the presence of intact anterior pituitary function or slight disturbance, especially when CT scanning detects curvilinear calcification, bony destruction and bony attachment, a sellar chondrosarcoma should be considered preferentially.

Calcification, heterogeneous enhancement, and impairments of visual acuity and visual field favor a sellar chondrosarcoma to be misdiagnosed as craniopharyngioma. Craniopharyngiomas are mainly located in the suprasellar cistern, often invading the hypothalamus and the third ventricle. Endocrine evaluations showed that about 70–90% patients with craniopharyngiomas present with a variety of anterior pituitary hormone deficiencies [13, 14]. In addition, diabetes insipidus is present in 23% of patients preoperatively [13]. The appearance of craniopharyngioma on MRI varies because they differ in the tumor consistency and in the content of the cyst fluid. On CT scans, the typical presentations of craniopharyngioma were nodular calcification with or without enlarged sella; bony destruction and bony attachment are rare [15]. Hence, the differentiation between sellar chondrosarcoma and craniopharyngioma is relatively easy based on a review of the clinical, endocrinological, and radiological data.

### Treatment outcomes

Surgical resection is the mainstream therapeutic modality for chondrosarcomas. Extent of resection, histological grade and adjuvant radiotherapy are known prognostic factors for both tumor recurrence and survival rate. In the pooled cases, the gross total resection rate was 58.3%, which is lower than that of chondrosarcomas occurring in other sites [3, 4, 16]. Sellar chondrosarcomas frequently extend into the cavernous sinus and involve cranial nerves and the internal carotid artery, which are considered to be associated with incomplete resection. Given the close relationship between residual tumor volume and histological grade and tumor recurrence, it is necessary to proceed to adjuvant therapy. Proton beam irradiation, gamma knife radiotherapy and intensity-modulated radiation therapy could yield good outcomes for patients with intracranial chondrosarcomas [17–21]. Furthermore, due to those lesions are radio-resistant, the adjuvant radio-enhancers can be effective in increasing the radiation damage on tumor cells while sparing surrounding brain parenchyma [20, 21]. Our 4 patients with grade I chondrosarcomas did not receive adjuvant radiotherapy due to the difficulty involved in identifying residual tumor component in postoperative MRI or achieving tumor complete resection. However, for patients with definite residual or recurrent tumor, we recommend postoperative radiotherapy. Owing to the small number of patients and the short follow-up duration, we could not evaluate the accurate progression free survival rate at 5 years.

## Conclusion

Chondrosarcoma is an unusual tumor entity of the sellar region, which can exhibit clinical, endocrine, and radiological features similar to other more common lesions in the region. The most common presentation is blurring of vision, followed by headache. Slight endocrine disorders occur in around 33.3% of such patients. The preoperative diagnosis of sellar chondrosarcoma remains challenging, and often requires differentiation from chordoma, INPA or craniopharyngioma. When a sellar calcified mass presents with intact or slightly disturbed anterior pituitary function, heterogeneous enhancement and no diffusion restriction on MRI sequences, surrounding bony destruction and bony attachment on CT scans, a chondrosarcoma should be suspected preferentially. Tumor complete resection is the optimal goal for management of sellar chondrosarcoma, but postoperative adjuvant radiotherapy and periodic follow-up should be highlighted.

## Data Availability

The analyzed data sets generated during the present study are available from the corresponding author on reasonable request.

## Abbreviations

EEA: Endonasal endoscopic approach
INPA: Invasive non-functioning pituitary adenoma
MRI: Magnetic Resonance Imaging
CT: Computed Tomography
EEES: Expanded Endoscopic Endonasal Surgery
